# Potential effectiveness of digital therapeutics specialized in executive functions as adjunctive treatment for clinical symptoms of attention-deficit/hyperactivity disorder: a feasibility study

**DOI:** 10.3389/fpsyt.2023.1169030

**Published:** 2023-07-20

**Authors:** Tai Hui Sun, Ji Won Yeom, Kwang-Yeon Choi, Jeong-Lan Kim, Heon-Jeong Lee, Hyun-Jin Kim, Chul-Hyun Cho

**Affiliations:** ^1^Department of Psychiatry, Korea University Anam Hospital, Seoul, Republic of Korea; ^2^Department of Psychiatry, Korea University College of Medicine, Seoul, Republic of Korea; ^3^Department of Psychiatry, Chungnam National University College of Medicine, Daejeon, Republic of Korea; ^4^Department of Biomedical Informatics, Korea University College of Medicine, Seoul, Republic of Korea

**Keywords:** attention-deficit/hyperactivity disorder, executive function, potential effectiveness, feasibility, digital therapeutics, adjunctive treatment, clinical symptoms

## Abstract

**Introduction:**

The role of digital therapeutics (DTx) in the effective management of attention deficit/hyperactivity disorder (ADHD) is beginning to gain clinical attention. Therefore, it is essential to verify their potential efficacy.

**Method:**

We aimed to investigate the improvement in the clinical symptoms of ADHD by using DTx AimDT01 (NUROW) (AIMMED Co., Ltd., Seoul, Korea) specialized in executive functions. NUROW, which consists of Go/No-go Task- and N-Back/Updating-based training modules and a personalized adaptive algorithm system that adjusts the difficulty level according to the user’s performance, was implemented on 30 Korean children with ADHD aged 6 to 12 years. The children were instructed to use the DTx for 15 min daily for 4 weeks. The Comprehensive attention test (CAT) and Childhood Behavior Checklist (CBCL) were used to assess the children at baseline and endpoint. In contrast, the ADHD-Rating Scale (ARS) and PsyToolkit were used weekly and followed up at 1 month, for any sustained effect. Repeated measures ANOVA was used to identify differences between the participants during visits, while t-tests and Wilcoxon signed-rank tests were used to identify changes before and after the DTx.

**Results:**

We included 27 participants with ADHD in this analysis. The ARS inattention (*F* = 4.080, *p* = 0.010), hyperactivity (*F* = 5.998. *p* < 0.001), and sum (*F* = 5.902, *p* < 0.001) significantly improved. After applying NUROW, internalized (*t* = −3.557, *p* = 0.001, 95% CI = −3.682-−0.985), other (*Z* = −3.434, *p* = 0.001, effect size = −0.661), and sum scores (*t* = −3.081, *p* = 0.005, 95% CI = −10.126-−2.022) were significantly changed in the CBCL. The overall effect was confirmed in the ARS sustained effect analysis even after 1 month of discontinuing the DTx intervention.

**Discussion:**

According to caregivers, the findings indicate that DTx holds potential effect as an adjunctive treatment in children with ADHD, especially in subjective clinical symptoms. Future studies will require detailed development and application targeting specific clinical domains using DTx with sufficient sample sizes.

**Clinical trial registration**: KCT0007579.

## Introduction

Attention-Deficit/Hyperactivity Disorder (ADHD) is one of the most common neurodevelopmental disorders that significantly impacts children’s lifestyles and performance in various settings, including home and school (1, 2). According to the Diagnostic and Statistical Manual of Mental Disorders: 5th Edition Text Revision (DSM-5-TR), the clinical presentation of ADHD can be predominantly inattentive, predominantly hyperactive/impulsive, and combined (1, 3). Patients with ADHD tend to prefer and seek for small, immediate rewards rather than large, temporally delayed rewards (4). Due to such changes, patients with ADHD struggle to focus and frequently distracted, making difficulties in maintaining appropriate academic performances and face problems with interpersonal relationships (5, 6). In addition, patients with ADHD may face comorbid mood and anxiety symptoms, which lead to behavioral changes such as depression, anxiety, and sleep disturbances, along with difficulties in social cognition, interpersonal skills, and low self-esteem (7–13). These ADHD symptoms can persist into adulthood in 60% of cases, making ADHD a chronic condition that burdens patients and caregivers (14). With approximately one in four children and adolescents receiving mental health services, raising children with ADHD may also result in psychological difficulties and conflicts (2, 15, 16).

Baseline treatment for ADHD involves pharmacological interventions, including stimulants and non-stimulants (17, 18). However, numerous risks are associated with using these medications, including increased heart rate and blood pressure and potential growth reduction (17). Also, there are still some concerns about the potential abuse risk of stimulants to children and adolescents with ADHD (19–21). In addition, medications are limited in their use as they can only be effective when administered properly (17, 22). There are several behavioral intervention options, including cognitive-behavioral therapy, behavioral parent training, and training teachers in classroom applications of contingency management techniques (23). However, issues, such as variation among behavioral therapists and the inability to attend all therapy sessions because of financial and time constraints, can be challenging for patients and their parents (24).

Digital therapeutics (DTx) have developed a new paradigm for ADHD treatment: easy therapeutic access, minimal side effects, and low risk of abuse or misuse to overcome such obstacles. DTx is a therapeutic intervention based on scientific evidence and powered by high-quality software programs for preventing, managing and treating medical disorders and diseases (25). It targets mental and behavior-modifiable conditions, including major mood disorders, anxiety disorders, substance use disorders, autism spectrum disorder, and ADHD (25–27). The FDA has approved EndeavorRx (Akili Interactive Labs, Boston, MA, United States), the first digital therapy indicated for ADHD (28). Several studies evaluating the efficacy of DTx targeting specific cognitive functions for the treatment of patients with ADHD have been conducted (29, 30).

A significant boost to the experiments and applications of telemedicine and DTx in clinical psychiatry has risen due to the pandemic. Using such telematics tools and DTx during the lockdown has provided potential in both the healthcare and social sectors, which can truly revolutionize healthcare system in the near future by integrating the treatments that has been already known. Since the pandemic, numerous studies and interests in these new digital approaches have been steadily increased examining their advantages and disadvantages, as well as prejudices and expectations of healthcare professionals and families toward these approaches (31–33). These efforts to develop innovative treatments tailored to individuals, respecting and trying to meet the needs of both the patients and health professionals as much as possible. Despite the increasing number of studies on DTx, trials are still in their early stages in clinical psychiatry. Although some DTx and smartphone applications target ADHD worldwide, there is a dearth of DTx, that targets the executive functions of ADHD (30, 34–36). In this study, we used AimDT01 (NUROW) (AIMMED Co., Ltd., Seoul, Korea), a prototype smartphone application developed to target executive functions in patients with ADHD, such as improving attention and working memory. NUROW, a combined word for “neuron” and “arrow,” was designed by a Korean healthcare company to target different fields of working memory by providing training modules in the form of video games. Each training module includes three distinct executive function domains: (i) the Go/No-go task to enhance sustained attention and inhibition control, (ii) the N-Back/Updating training module to improve working memory, and (iii) the memorization training technique to improve attention in participants with ADHD. NUROW is expected to improve the clinical need for adjuvant treatment options, given that behavioral intervention, and pharmacological treatment are essential for providing overall benefits to participants with ADHD (35, 37). The objective of this study was to assess the potential effectiveness of DTx (NUROW) in treating clinical symptoms of ADHD in children. The evaluation involved measuring changes in (1) subjective clinical symptoms, including ARS and CBCL, and (2) objective neuropsychological domains, such as CAT and PsyToolkit.

## Method

### Participants and study design

Participants aged 6 to 12 years were recruited from the outpatient clinic of the Chungnam National University Sejong Hospital. Patients were recruited from hospital advertisements and online flyers, and the study was conducted from December 2020 to February 2022. During the medical examination and treatment in the psychiatric outpatient clinic, if the patient is considered a potential candidate to participate in the study, the clinician has recommended the patients and their caregivers participate in the study. A clinician (HJK) screened 35 participants and diagnosed children with ADHD using the Kiddie Schedule for Affective Disorders and Schizophrenia Present and Lifetime Version (38).

**Figure 1:**
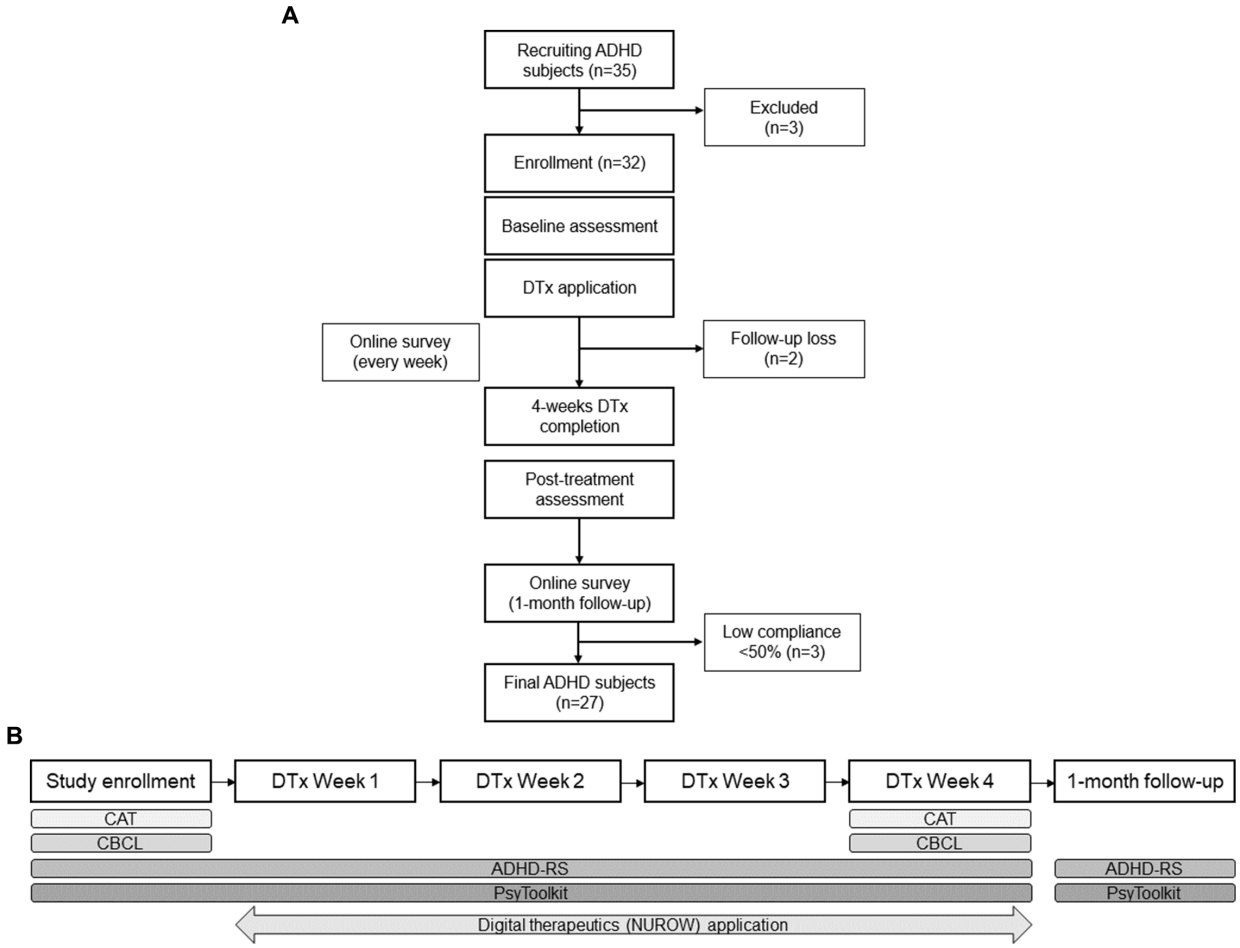
Schematic research flow of using digital therapeutic (NUROW) on Korean children with ADHD **(A)** Overall study design of digital therapeutics application and participant flow. **(B)** Neuropsychological assessment framework for evaluating effects of digital therapeutics in participants with ADHD. ADHD, Attention Deficit/Hyperactivity Disorder; DTx, digital therapeutics; CAT, Comprehensive Attention Test; CBCL, Childhood behavioral checklist; ARS, ADHD-Rating Scale.

Overall study design is presented in Figure 1. Participants taking ADHD medications were not permitted to alter their dosage for 2 months to control for any potential pharmacological effects during the study. Children taking antidepressants, antipsychotics, or anxiolytics were excluded from the study due to the possibility of neurocognitive side effects that could alter the baseline. Participants with intellectual disabilities, autism spectrum disorders, organic brain diseases, congenital disorders, or any incurable neurological conditions, including seizures or sensory disorders, were excluded. After screening, 30 participants with ADHD were enrolled in the study. During the study, 2 more participants were dropped out due to follow-up loss, leaving with final 30 participants to complete the study. Furthermore, participants who did not meet the standard recommended usage time of NUROW, specifically less than 50%, were considered noncompliant and therefore excluded from the analysis.

After a thorough explanation and comprehension of the study protocol, all participants and their caregivers provided written consent after full explanation and understanding of the study protocol. During the study, no participants and their caregivers received any financial assistance (e.g., cash). However, neuropsychological tests for assessing the participants were provided free during the study. The Institutional Review Board approved this study and was registered with the Clinical Research Information Service (KCT0007579).

### Digital therapeutic interventions (AimDT01, NUROW)

NUROW is a preliminary trial product of a digital therapeutic smartphone application specializing in executive functions, such as attention and working memory. It was developed by the Korean company AIMMED Co., Ltd., in collaboration with psychiatrists and cognitive science specialists. It aims to improve the ability to pay attention for a long duration, avoid distraction, and focus on multiple signals simultaneously.

NUROW is compatible with the iOS and Android operating systems. In this study, the participants were instructed to use NUROW for 15 min daily, 5 days weekly, for 4 successive weeks. Prior to the study, participants were required to log into the NUROW system using their parents’ e-mail addresses or social network services accounts. After filling in the information of the participant’s name, gender, date of birth, and school grade level, the participants’ caregivers were given the option to click on ADHD symptom-related questionnaires and the participant’s academic achievement level. In addition to personal information, application usage log data, including the date and duration of use, were collected for 4 weeks.

Each of the three main training modes in NUROW is named after one of the three main characters in the novel *Journey to the West* (*Sun Wukong or Monkey King*, *Zhu Bajie or Pigsy*, *Sha Wujing or Sandy*), each with a tutorial session. *Journey to the West* is a Chinese novel by Wu Cheng and published in the 16th century. It is regarded as one of East Asia’s most classic Chinese novels and a well-known character-driven story (39).

Each training module was designed to develop distinct working memory domains. The participants were permitted to choose any of the three main training modules and were instructed to spend at least 15 min daily on any training module (Supplementary Figure S1). The *Sun Wukong or Monkey King* training mode targets sustained attention and inhibition control using the Go/No-go Task. The user is instructed to tap the screen (catching a fist) when a typical signal appears and persists until another signal appears. The *Zhu Bajie or Pigsy* training mode is an N-Back/Updating-based training module designed to improve working memory by asking the participant to count the number of times a specific character appears on the screen while playing the pressing game. Finally, *Sha Wujing or Sandy* mode requires the user to memorize the sequence in which the characters appear on the screen, which aims to improve attention.

A real-time reflection of the user’s difficulty level in each training module is enabled by an adaptive algorithm system in NUROW. NUROW automatically adjusts the difficulty level of each training session based on the user’s performance at a certain level. For example, if a user feels a level is familiar and easy, NUROW shortens the response time, making the level relatively difficult. Parents were notified by phone calls if the NUROW was not used for 48 consecutive hours and on the 28^th^ (4^th^ week) of study participation at the end of the study.

## Assessment

### Attention deficit/hyperactivity disorder-Rating Scale

Parents of children with ADHD were requested to complete the ARS online. ARS was assessed weekly from baseline to the endpoint and at the 1-month follow-up. The ARS is one of the commonly used ADHD evaluation scales (40, 41). The ARS was based on the diagnostic criteria for ADHD, consisting of 18 questionnaires assessing for inattention and hyperactivity/impulsivity. The ARS yielded three domains: inattention, hyperactivity, and sum scores. High ARS scores indicated a diminished ability for attention and impulse control.

### Childhood Behavior Checklist

The CBCL is a standardized checklist for parents to assess child behavioral problems. The CBCL was assessed twice at the beginning and end of the study. The CBCL consists of questionnaires regarding children’s behavior in the previous 6 months and combines them into nine subscales for behavioral problem. Internalizing (e.g., anxiety, withdrawal, and somatic symptoms) and externalizing (e.g., aggression and rule-breaking) behaviors are included in CBCL questionnaires. In addition, the CBCL includes mood, anxiety, and somatic disorders (42).

### Computerized Comprehensive Attention Test

Using the computerized CAT developed by the Korean Academy of Child and Adolescent Psychiatry, various measures of attention were evaluated (43). CAT is a modified continuous-performance assessment for Korean children and adolescents that uses auditory and visual stimuli. The CAT has six subscales: visual selective, auditory selective, sustained attention to the response, Flanker’s task/effect, divided attention, and working memory.

The omission error, commission error, mean reaction time, and standard deviation (SD) of reaction time (response time variability) were measured for each subscale (44). CAT was supervised by a well-trained clinical psychologist and was assessed at the beginning and end of treatment. In sustained attention, participants were asked to respond to all shape stimuli except the “x” shape to measure their ability to inhibit responses while other stimuli were presented. Auditory and visual stimuli were presented to participants every 2 s for 3 min 20 s. They were instructed to respond only to the same pair of stimuli as before to measure divided attention.

### PsyToolkit

PsyToolkit is an open-access experimental service for conducting cognitive and psychological laboratory and internet-based experiments and surveys (45). In this study, cognitive neuropsychological tests, such as the Corsiblock, Stroop test, Simon’s task, and Wisconsin card sorting test (WCST), were conducted via web-based version to measure reaction time and error rate. Each participant with ADHD used PsyToolkit from baseline to endpoint weekly and 1 month after digital therapeutic intervention.

Corsiblock aims to assess spatial short-term working memory by instructing the user to memorize a sequence of flashing blocks and click them in the same order (46). The Stroop test is a popular neuropsychological measure of executive function, selective attention, cognitive flexibility, and processing speed (47). Participants are asked to identify the color of the printed material of the given color word. Simon’s task is a well-established neuropsychological test that evaluates the speed and accuracy of response to various stimuli (48). The WCST is a widely used neuropsychological test for evaluating executive function (49).

### Statistical analyzes

The primary objective of this study was to investigate the potential effect of NUROW on subjective clinical symptoms through the assessment of ARS and CBCL. The secondary objective was to examine the potential effect on objective neuropsychological domains through the assessment of CAT and PsyToolkit. ARS and PsyTookit results were analyzed using repeated-measures analysis of variance (ANOVA) with within-participants visit factors. According to the normality test, Wilcoxon’s signed-rank tests or paired *t*-tests were conducted on the CBCL and CAT. Wilcoxon’s signed-rank test or paired *t*-test were used to analyze potential sustained effect of NUROW by ARS and PsyToolkit between the endpoint and 1 month later. All analyzes results were considered statistically significant when *p* < 0.05. IBM SPSS Statistics (version 26.0; SPSS Inc., Chicago, IL, United States) was used to analyze data.

## Result

To ensure reliable analysis, we calculated the application log information regarding the usage time. We carefully reviewed the total usage time for each child throughout the study and identified participants who demonstrated poor compliance, defined as having a total usage time below 50% of the standard recommended usage time for NUROW. Three children were categorized as poor compliant participants. The remaining 27 children with ADHD (90% of the total subjects) were shown to have played NUROW compliantly. Table 1 presents the demographic and primary clinical characteristics of the participants. In this study, patients with ADHD were found to be taking methylphenidate and atomoxetine at mean dosages of 28.89 mg and 28.13 mg, respectively.

**Table 1:** Demographic and clinical characteristics of participants with attention-deficit/hyperactivity disorder (ADHD) (*n* = 27) at baseline.

Variable	
Gender, *n* (%)	
Female	5 (18.52)
Male	22 (81.48)
Age, mean (SD), y	8.11 (1.60)
Age, range, y	6–12
Height, mean (SD), cm	133.28 (11.95)
Weight, mean (SD), kg	33.08 (11.38)
Ethnicity, *n* (%)	
Asian (Korean)	27 (100)
ADHD medication, *n* (%)	
Methylphenidate	20 (74.07)
Average dose, mg	28.89
Atomoxetine	7 (25.92)
Average dose, mg	28.13
Clinical measures	Mean (SD)
ADHD-Rating Scale	
Inattention	20.22 (5.639)
Hyperactivity	19.59 (6.28)
Sum	39.81 (11.48)
PsyToolkit	
Corsiblock	3.30 (2.334)
Stroop test	
Congruent reaction time	1110.83 (257.52)
Incongruent reaction time	1142.69 (335.98)
Compatible error rate	39.69 (32.15)
Incompatible error rate	28.79 (28.71)
Simon task	
Congruent reaction time	1570.03 (500.58)
Incongruent reaction time	1750.62 (644.84)
Compatible error rate	21.23 (18.44)
Incompatible error rate	20.90 (19.83)
Wisconsin card sorting test	
Total error rate	28.40 (9.94)
Perseverative error	18.46 (5.90)
Non-perseverative error	9.94 (5.67)

SD, standard deviation.

### Attention deficit/hyperactivity disorder-Rating Scale

**Figure 2:**
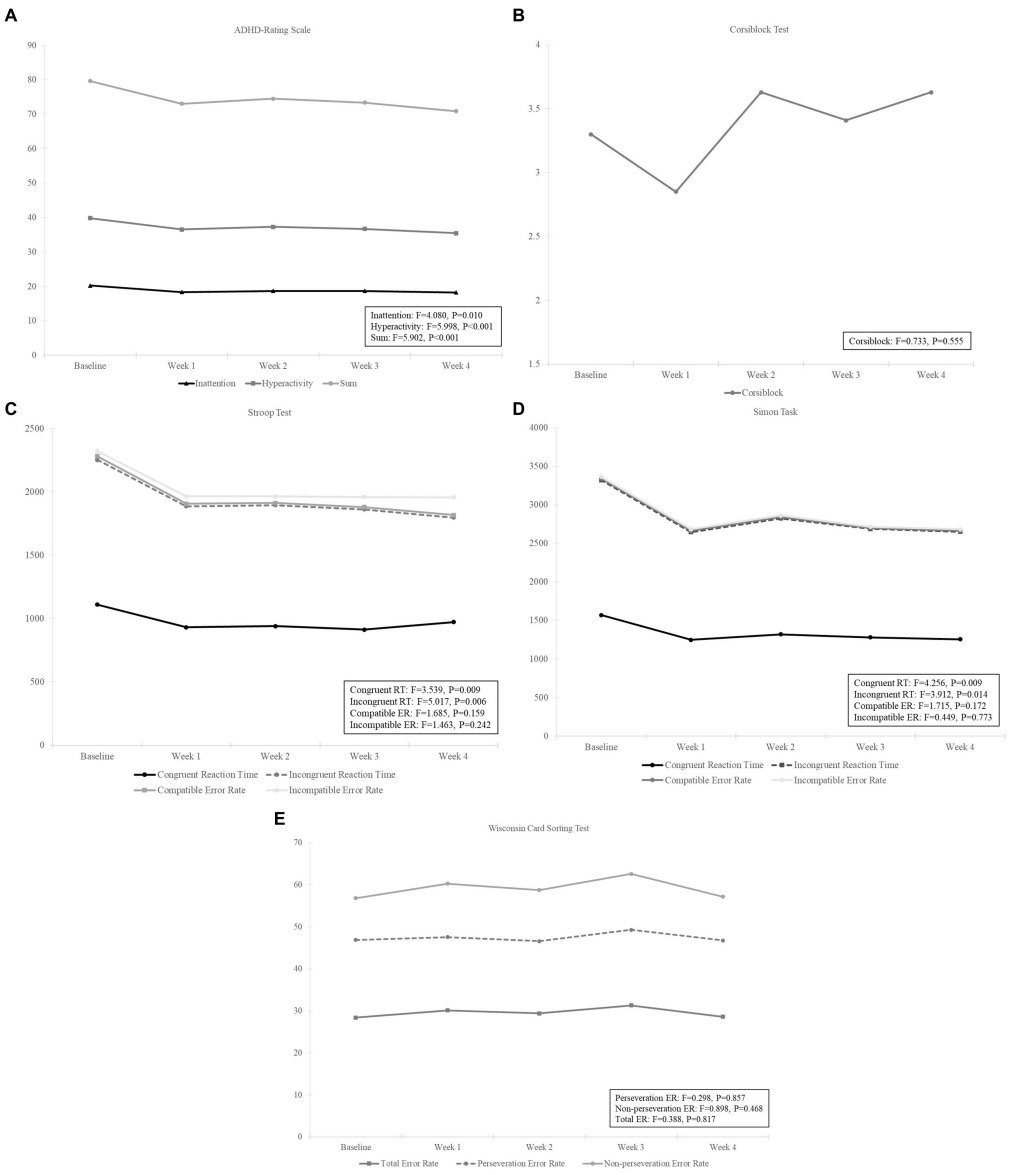
Results using a repeated measures analysis of variance on **(A)** ADHD-Rating Scale, **(B)** Corsiblock Test, **(C)** Stroop Test, **(D)** Simon Task, and **(E)** Wisconsin Card Sorting Test measures in patients with ADHD during digital therapeutics (NUROW) intervention. CRT, Congruent Reaction Time; IRT, Incongruent Reaction Time; CER, Compatible Error Rate; IER, Incompatible Error Rate; TER, Total Error Rate; PER, Perseveration Error Rate; NER, Non-Perseveration Error Rate.

In this study, repeated-measures ANOVA was performed to determine differences in ARS within hospital visits. Mean(±SD) scores of ARS inattention in the baseline and endpoint were 20.22 (±5.639) and 18.26 (±5.34), respectively, with statistically significant difference (*F* = 4.080, *p* = 0.010). Mean (±SD) scores of ARS hyperactivity in the baseline and endpoint were 19.59 (±6.28) and 17.19 (±6.06), respectively, with statistically significant difference (*F* = 5.998, *p* < 0.001). Mean (±SD) score of ARS sum scores in the baseline and endpoint were 39.81 (±11.48) and 35.44 (±11.04), respectively, showing a significant decrease after DTx intervention (*F* = 5.902, *p* < 0.001; Table 2; Figure 2).

**Table 2:** Result of a repeated measures analysis of variance on the differences of ADHD-Rating Scale and PsyToolkit measures in patients with ADHD during digital therapeutics (NUROW) intervention.

Measures	Baseline, mean (SD)	Week 1, mean (SD)	Week 2, mean (SD)	Week 3, mean (SD)	Week 4, mean (SD)	F statistic (df)	*p* value
ARS							
Inattention	20.22 (5.639)	18.33 (5.51)	18.67 (5.26)	18.67 (5.16)	18.26 (5.34)	4.080 (2.987)	0.010
Hyperactivity	19.59 (6.28)	18.19 (5.92)	18.59 (5.87)	18.00 (6.52)	17.19 (6.06)	5.998 (4)	<0.001
Sum	39.81 (11.48)	36.52 (10.96)	37.26 (10.73)	36.67 (11.17)	35.44 (11.04)	5.902 (3.595)	<0.001
PsyToolkit							
Corsiblock	3.30 (2.334)	2.85 (2.196)	3.63 (2.115)	3.41 (1.947)	3.63 (2.169)	0.733 (3.497)	0.555
Stroop test							
Congruent RT	1110.83 (257.52)	931.26 (318.41)	940.53 (234.28)	913.46 (296.50)	973.31 (250.11)	3.539 (4)	0.009
Incongruent RT	1142.69 (335.98)	956.03 (291.08)	955.87 (279.00)	948.06 (335.83)	824.25 (342.00)	5.017 (2.498)	0.006
Compatible ER	28.79 (28.71)	19.69 (27.18)	16.97 (25.28)	19.65 (25.18)	19.61 (18.33)	1.685 (4)	0.159
Incompatible ER	39.69 (32.15)	57.14 (137.92)	50.65 (140.40)	79.43 (220.58)	140.31 (353.46)	1.463 (1.741)	0.242
Simon task							
Congruent RT	1570.03 (500.58)	1248.97 (503.35)	1319.25 (512.16)	1281.21 (456.48)	1255.73 (360.59)	4.256 (2.892)	0.009
Incongruent RT	1750.62 (644.84)	1397.81 (583.45)	1504.57 (623.95)	1405.60 (482.82)	1395.57 (412.14)	3.912 (2.782)	0.014
Compatible ER	21.23 (18.44)	17.09 (19.72)	18.19 (16.86)	14.47 (14.16)	12.38 (13.01)	1.715 (2.943)	0.172
Incompatible ER	20.90 (19.83)	19.75 (23.49)	18.24 (17.50)	14.93 (14.49)	18.75 (16.62)	0.449 (4)	0.773
WCST							
Total ER	28.40 (9.94)	30.12 (10.17)	29.38 (10.09)	31.30 (14.32)	28.58 (10.53)	0.388 (4)	0.817
Perseveration ER	18.46 (5.90)	17.41 (4.86)	17.22 (4.16)	17.96 (5.91)	18.15 (4.99)	0.298 (3.528)	0.857
Non-perseveration ER	9.94 (5.67)	12.72 (6.94)	12.16 (9.33)	13.33 (12.33)	10.43 (6.99)	0.898 (4)	0.468

SD, standard deviation; df, degrees of freedom; ARS, ADHD-Rating Scale; RT, Reaction time; ER, Error rate; WCST, Wisconsin Card Sorting Test.

### Childhood Behavior Checklist

Since we analyzed a total of 11 subdomains for CBCL Problem and 8 subdomains for CBCL DSM-5 Diagnosis, we applied Bonferroni correction for multiple comparisons. Therefore, the statistical significance of the analysis results for subdomains (excluding Sum) of CBCL Problem was *p* < 0.0045 (0.05/11), and the statistical significance of the analysis results for subdomains of CBCL DSM-5 Diagnosis was *p* < 0.0063 (0.05/8). For CBCL Problem, internalized (*t* = −3.557, *p* = 0.001, 95% CI = −3.682-−0.985), other (*Z* = –3.434, *p* = 0.001, effect size = −0.661), and sum scores (*t* = −3.081, *p* = 0.005, 95% CI = −10.126-−2.022) were significantly changed after applying NUROW. Regarding the CBCL DSM-5 diagnosis, no subdomain showed a significant change after using DTx (Table 3).

**Table 3:** Changes in Childhood behavior checklist (CBCL) and Computerized Comprehensive Attention Test (CAT) measures in children with ADHD using digital therapeutics (NUROW).

Measures		Mean (SD)	*t* (df) or Z	*p* value	95% CI or Effect size
CBCL problem					
Anxiety depression	Pre-treatment	5.37 (3.753)	−2.482 (26) *	0.020	−2.167– −0.204
	Post-treatment	4.19 (3.420)			
Withdrawal depression	Pre-treatment	2.19 (2.149)	−1.052†	0.293	−0.202
	Post-treatment	1.81 (1.711)			
Somatic	Pre-treatment	1.96 (2.295)	−2.051†	0.040	−0.395
	Post-treatment	1.19 (1.331)			
Social	Pre-treatment	5.78 (3.389)	−1.828†	0.068	−0.352
	Post-treatment	4.81 (3.363)			
Thought	Pre-treatment	3.19 (2.512)	−0.980†	0.327	−0.189
	Post-treatment	3.07 (3.304)			
Attention	Pre-treatment	8.00 (3.340)	−1.667 (26) *	0.108	−1.903–0.199
	Post-treatment	7.15 (3.072)			
Rule breaking	Pre-treatment	3.48 (2.806)	−1.799†	0.072	−0.346
	Post-treatment	2.85 (2.957)			
Aggression	Pre-treatment	9.52 (7.623)	−1.515 (26) *	0.142	−3.230–0.489
	Post-treatment	8.15 (7.363)			
Other	Pre-treatment	5.19 (2.909)	−3.434†	0.001	−0.661
	Post-treatment	3.89 (2.636)			
Internalized	Pre-treatment	9.52 (5.873)	−3.557 (26) *	0.001	−3.682–−0.985
	Post-treatment	7.19 (4.985)			
Externalized	Pre-treatment	13.00 (9.774)	−1.755 (26) *	0.091	−4.343–0.343
	Post-treatment	11.00 (9.992)			
Sum	Pre-treatment	35.67 (18.679)	−3.081 (26) *	0.005	−10.126–−2.022
	Post-treatment	29.59 (18.327)			
CBCL DSM-5 diagnosis					
Emotion	Pre-treatment	3.48 (2.833)	−0.602†	0.547	−0.116
	Post-treatment	3.19 (2.512)			
Anxiety	Pre-treatment	2.96 (1.870)	−2.966†	0.003	−0.571
	Post-treatment	2.07 (1.920)			
Somatic	Pre-treatment	0.93 (1.412)	−2.070†	0.038	−0.398
	Post-treatment	0.48 (0.700)			
Attention deficit/hyperactivity disorder	Pre-treatment	7.56 (3.080)	−1.922 (27) *	0.066	−2.069–0.069
	Post-treatment	6.56 (3.286)			
Oppositional defiant disorder	Pre-treatment	3.52 (2.242)	−1.209†	0.227	−0.233
	Post-treatment	3.04 (2.278)			
Conduct disorder	Pre-treatment	3.85 (4.130)	−1.119†	0.263	−0.215
	Post-treatment	3.52 (4.136)			
Obsessive–compulsive disorder	Pre-treatment	2.52 (1.988)	−2.219 (27) *	0.035	−1.712–−0.065
	Post-treatment	1.63 (1.690)			
Posttraumatic stress disorder	Pre-treatment	7.22 (3.613)	−2.766 (27) *	0.010	−2.389–−0.352
	Post-treatment	5.85 (3.955)			
CAT–visual selective attention					
Omission error	Pre-treatment	15.07 (18.58)	−0.377†	0.706	
	Post-treatment	14.44 (17.05)			
Commission error	Pre-treatment	29.30 (29.25)	−0.832 (26) *	0.413	−12.596–5.336
	Post-treatment	25.67 (21.33)			
Reaction time	Pre-treatment	29.30 (29.25)	−2.442 (26) *	0.022	−65.748–−5.651
	Post-treatment	505.59 (86.24)			
Reaction time-SD	Pre-treatment	212.54 (121.89)	−0.120†	0.904	
	Post-treatment	211.06 (103.95)			
CAT–auditory selective attention					
Omission error	Pre-treatment	14.59 (15.43)	−1.436†	0.151	−0.072
	Post-treatment	20.19 (22.83)			
Commission error	Pre-treatment	25.19 (29.01)	−0.508†	0.611	−0.023
	Post-treatment	23.78 (18.23)			
Reaction time	Pre-treatment	672.44 (140.65)	−1.481 (26) *	0.151	−79.207–12.857
	Post-treatment	639.27 (125.65)			
Reaction time-SD	Pre-treatment	273.69 (95.26)	0.174 (26) *	0.863	−34.420–40.806
	Post-treatment	276.89 (109.64)			
CAT–sustained attention to response					
Omission error	Pre-treatment	31.07 (37.67)	−1.952†	0.051	−0.376
	Post-treatment	46.30 (47.09)			
Commission error	Pre-treatment	22.41 (11.98)	0.513 (26) *	0.612	−3.900–6.492
	Post-treatment	23.70 (11.89)			
Reaction time	Pre-treatment	630.93 (105.89)	0.555 (26) *	0.584	−34.300–59.673
	Post-treatment	643.62 (127.16)			
Reaction time-SD	Pre-treatment	274.61 (137.09)	−0.841†	0.400	−0.162
	Post-treatment	275.00 (102.34)			
CAT–Flanker’s test					
Omission error	Pre-treatment	20.00 (21.19)	0.531 (26) *	0.600	−6.587–11.180
	Post-treatment	22.30 (25.44)			
Commission error	Pre-treatment	28.44 (22.28)	−2.869 (26) *	0.008	−17.101–−2.825
	Post-treatment	18.48 (12.29)			
Reaction time	Pre-treatment	702.24 (180.67)	1.608 (26) *	0.120	−10.258–83.951
	Post-treatment	739.09 (179.94)			
Reaction time-SD	Pre-treatment	274.63 (126.25)	−1.032 (26) *	0.312	−60.612–20.103
	Post-treatment	254.37 (117.49)			
CAT–divided attention					
Omission error	Pre-treatment	16.70 (9.61)	−1.840†	0.066	−0.354
	Post-treatment	14.96 (10.30)			
Commission error	Pre-treatment	16.93 (12.22)	−0.381†	0.703	−0.073
	Post-treatment	15.11 (8.03)			
Reaction time	Pre-treatment	731.79 (180.96)	0.828 (26) *	0.415	−43.582–102.400
	Post-treatment	761.19 (146.70)			
Reaction time-SD	Pre-treatment	332.08 (95.10)	−1.498 (26) *	0.146	−67.049–10.522
	Post-treatment	303.82 (73.67)			
CAT–spatial working memory					
Forward correct response	Pre-treatment	4.07 (1.21)	−1.590†	0.112	−0.306
	Post-treatment	4.48 (1.12)			
Forward memory span	Pre-treatment	5.19 (2.18)	0.416 (26) *	0.681	−0.704–1.061
	Post-treatment	5.37 (1.86)			
Backward correct response	Pre-treatment	3.40 (1.90)	−2.720†	0.007	−0.523
	Post-treatment	4.41 (1.58)			
Backward memory span	Pre-treatment	4.02 (2.78)	2.954 (26) *	0.007	0.479–2.670
	Post-treatment	5.59 (2.42)			

SD, standard deviation; CI, Confidence interval; CBCL, Childhood Behavior Checklist; CAT, Comprehensive Attention Test. *The following results were obtained using *t*-tests. †The following results were obtained using Wilcoxon Signed-rank tests. Bonferroni correction was used for multiple comparisons since we investigated 11 subdomains for CBCL Problem and 8 for DSM-5 Diagnosis. Thus, the statistically significant results for subdomains (excluding Sum) of CBCL Problem were *p* < 0.0045 (0.05/11) and *p* < 0.0063 (0.05/8) for DSM-5 Diagnosis.

### Computerized CAT

The results showed that there were no statistically significant differences in the omission and commission errors of selective visual attention and auditory selective attention, between the baseline and endpoint. However, there was a significant difference in the mean reaction time of selective visual attention between the baseline and endpoint (*t* = −2.422, *p* = 0.022). The sustained attention to response showed no significant differences in omission and commission errors, along with reaction time and reaction time SD between the baseline and endpoint. The Flanker’s test showed a significant difference in the commission errors between the baseline and endpoint (*t* = −2.869, *p* = 0.008), but no significant differences in omission errors, reaction time, and reaction time SD. There was no significant difference between the baseline and endpoint in divided attention in terms of omission and commission errors and reaction time and reaction time SD. For spatial working memory measure, 4 sub-items were measured: correct forward response, forward memory span, backward correct response, and backward memory span. Only backward correct response (*Z* = –2.720, *p* = 0.007) and backward memory span (*t* = 2.954, *p* = 0.007) were improved after DTx, with statistical significance (Table 2; Figure 2).

### PsyToolkit

For Corsiblock, there was no significant difference between visits (*F* = 0.773, *p* = 0.555). In the Stroop test, congruent reaction time and incongruent reaction time were significantly different within the visits [congruent (*F* = 3.539, *p* = 0.009) and incongruent (*F* = 5.017, *p* = 0.006)], while there was no significant difference within the visits for error rate [compatible error rate (*F* = 1.685, *p* = 0.159) and incompatible error rate (*F* = 1.463, *p* = 0.242)]. For the Simon task, congruent and incongruent reaction times were significantly different within the visits [congruent (*F* = 4.256, p = 0.009) and incongruent (*F* = 3.912, *p* = 0.014)], while there was no significant difference within the visits for error rate [compatible error rate (*F* = 1.715, *p* = 0.172), incompatible error rate (*F* = 0.449, *p* = 0.773]). In the WCST, there was no significant difference in the hospital visits for total error rate (*F* = 0.388, *p* = 0.817), perseveration error rate (*F* = 0.298, *p* = 0.857), or non-perseveration error rate (*F* = 0.898, *p* = 0.468). All mean values and error rates are listed in Table 2 and Figure 2. We speculated that NUROW has a potential effect on reducing reaction times in patients with ADHD. However, PsyToolkit’s analysis could not confirm the clinically meaningful and interpretable results.

### Sustained effect: 1-month follow-up

Sustained effects in ARS and PsyToolkit were investigated between the endpoint of the digital therapeutic session and 1 month later. For ARS, 1-month follow-up mean(±SD) scores for inattention, hyperactivity, and sum were 17.81(±5.55), 17.22(±6.59), and 35.04(±11.56), respectively (Table 4), while there was no significant difference in all the three measures (Inattention: *t* = −1.210, *p* = 0.237; Hyperactivity: *Z* = −0.020, *p* = 0.984, Sum: *t* = −0.942, *p* = 0.346), suggesting a potential, clinically meaningful sustained effect after the discontinuation of NUROW.

**Table 4:** Results of comparative analysis of ADHD-Rating Scale and PsyToolkit neuropsychological measures between immediately after digital therapeutic intervention (Week 4) and follow-up after 1 month to confirm the sustained effect of digital therapeutics (NUROW).

Measure	Week 4, Mean (SD)	1-month follow up, Mean (SD)	t (df) or Z	P value
ARS				
Inattention	18.26 (5.34)	17.81 (5.55)	−1.210 (26) *	0.237
Hyperactivity	17.19 (6.06)	17.22 (6.59)	−0.020†	0.984
Sum	35.44 (11.04)	35.04 (11.56)	−0.942†	0.346
PsyToolkit				
Corsiblock	3.63 (2.17)	3.37 (1.884)	−0.475†	0.634
Stroop test				
Congruent RT	973.31 (250.11)	957.10 (236.68)	−0.714†	0.475
Incongruent RT	824.25 (342.00)	846.93 (365.61)	0.560 (26) *	0.580
Compatible ER	20.40 (25.37)	20.36 (18.26)	−0.483†	0.629
Incompatible ER	140.31 (353.46)	124.02 (318.62)	−0.600†	0.549
Simon task				
Congruent RT	1255.73 (360.59)	1206.91 (464.60)	−1.278†	0.201
Incongruent RT	1395.67 (412.14)	1384.42 (470.05)	−0.013†	0.989
Compatible ER	12.38 (13.01)	13.09 (18.68)	0.257 (26) *	0.799
Incompatible ER	18.75 (16.62)	12.52 (17.11)	−2.127†	0.033
WCST				
Total ER	28.58 (10.53)	29.20 (12.09)	0.280 (26) *	0.782
Perseveration ER	18.15 (4.99)	16.23 (5.06)	−1.841†	0.066
Non-perseveration ER	10.43 (6.99)	12.96 (8.21)	−1.461†	0.144

SD, Standard deviation; df, degrees of freedom; ARS, ADHD-Rating Scale; RT, Reaction time; ER, Error rate; WCST, Wisconsin Card Sorting Test. *The following results were obtained using t-tests. †The following results were obtained using Wilcoxon Signed-rank tests.

In the Corsiblock test of PsyToolkit, the 1-month follow-up mean(±SD) score was 3.37(±1.88), with no significant difference (*Z* = -0.475, *p* = 0.634). For the Stroop task of PsyToolkit, the 1-month follow-up mean(±SD) score of congruent reaction time, incongruent reaction time, compatible error rate, and incompatible error rate were 957.10(±236.68), 846.93(±365.61), 20.36(±18.26) and 124.02(±318.62), respectively, with no statistically significant difference. In the Simon task of PsyToolkit, 1-month follow-up mean(±SD) scores of congruent reaction time, incongruent reaction time, compatible error rate, and incompatible error rate were 1206.91(±464.60), 1384.42(±470.05), 13.09(±18.68), and 12.52(±17.11), respectively, showing a significant difference only in the incompatible error rate 1 month after applying DTx (Z = -2.127, *p* = 0.003). For WCST, the 1-month follow-up mean(±SD) score of total error rate, perseveration error rate, and non-perseveration error rate were 29.20(±12.09), 16.23(±5.06), and 12.96(±8.21), respectively. No statistically significant difference was found for the WCST, suggesting possible sustained effect.

## Discussion

This study indicates that NUROW significantly improved our primary outcome measure, a clinical symptom of ADHD, especially inattentiveness and hyperactivity. To the best of our knowledge, this is the first study to evaluate the feasibility and possible clinical effects of adjunctive DTx for individuals with ADHD in South Korea. Our results are consistent with previous studies involving other DTx for ADHD (29, 30, 35, 50–53). ARS is a parent-reported measure; it indicates that caregivers of children with ADHD subjectively experienced positive changes in their child’s behavior after using NUROW. The CBCL, another parent-reported measure, also indicated a significant difference after using DTx. After using NUROW, anxiety, depression, somatic, internalizing, additional problems, and total CBCL scores declined in this study. Although the participants’ diagnoses other than ADHD were not confirmed in this study, fidgeting and restlessness in the behavior of children with ADHD may have overlapped with anxiety items in the CBCL (6, 9, 10, 54, 55), indicating that the use of DTx helped behavioral aspects of ADHD. For somatic symptoms on the CBCL, it is known that children with ADHD have comorbidities of somatic disorders (6, 56). Our findings indicate the potential efficacy of DTx in reducing subjective ADHD symptoms, despite the absence of financial assistance for participants and their caregivers in the current study. NUROW may be an adjunctive treatment option for some of the difficulties faced by current interventions, such as medications and behavioral therapy. During the study, no adverse effects of NUROW, including dizziness, nausea, or headache, were observed. Furthermore, as part of a feasibility study, the current research classified participants based on their usage time compared to the recommended usage time, with 90% of the participants demonstrating reasonable compliance (over 50% of the recommended usage time) until the completion of the study. Hence, NUROW was regarded as a feasible digital therapeutic option for individuals with ADHD.

Although it was possible to confirm a significant improvement trend following DTx intervention in the clinical and behavioral domains (ARS and CBCL), there was insufficient evidence that NUROW had a significant change on the neurocognitive domains (CAT and PsyToolkit). Although the NUROW applied in this study was developed specifically for executive function, the lack of a significant therapeutic effect in the neurocognitive domain is a topic that requires further investigation. Various studies on the psychiatric application of serious games, including DTx, have been conducted recently; in particular the potential of DTx in improving neurocognitive symptoms in ADHD, major neurocognitive disorder, and depression (26–30). However, it seems too early to draw a conclusion on the cognitive function improvement effect in this study. Although there was no significant effect on the neurocognitive domain, some attention-related measures (e.g., reaction time) from PsyToolkit showed statistically significant improvements when DTx was used. It is well-known that a learning effect occurs when cognitive tests are repeated routinely (56, 57). From the results of this study, PsyToolkit was assessed weekly, and computerized CAT was assessed at the baseline and endpoint. Although cognitive tests were performed repeatedly, there was no significant effect on cognitive tasks. Only the incompatible error rate sub-item of Simon’s task in PsyToolkit decreased with statistical significance. To interpret these results, we must understand the study’s design methodology. Although potential confounding factors, such as psychiatric medications, were regulated to the greatest extent, the study design and sample size were insufficient to accurately evaluate the direct effects of DTx on the neurocognitive domain. Nevertheless, the ability to confirm the potential effect in the clinical and behavioral domains is an encouraging result, and it will serve as the foundation for future research and development of DTx.

Clinically, it is essential to confirm whether there is a sustained effect after treatment. In this regard, the study’s confirmation of the sustained effect even after 1 month is an additional strength. ARS, a measure that confirmed the potential therapeutic efficacy of NUROW by evaluating the level of clinical improvement at the end of the intervention and 1 month later, showed no statistical difference between the two-time points. This has positive implications for the potential long-term effectiveness of NUROW, particularly in clinical and behavioral domains. Our study results have found that there were other domains, such as Corsiblock test, Stroop task, and WCST, found to have sustained effect of DTx even after 1 month of discontinuation. These results suggest that DTx has also showed possibility of persistent effects on neurocognitive domains. However, as previously mentioned, it would be limited to discuss the sustained effect in terms of PsyToolkit because it was difficult to confirm its clear impact of PsyToolkit during DTx intervention. A feasibility study of DTx in multiple sclerosis patients explored both the therapeutic and sustained effects of DTx (58). Several previous studies have examined the efficacy of DTx in treating ADHD. However, none have examined whether DTx has a sustained effect after discontinuation, to our knowledge. Recent studies have focused on the efficacy of digital therapeutics (DTx) when in use, leaving the effects of sustained use largely unexplored (29, 30, 35, 50, 51, 53). Our study aims to address this gap, investigating the possible effects of DTx and the potential for sustained effects even after the participants have stopped using the DTx. This will allow us to gain a comprehensive understanding of the potential effects of DTx and how they might be sustained. We believe that our findings could open up new possibilities for the use of DTx in the future.

However, there are certain limitations to this study. First, this study was conducted with a relatively small sample size; however, it has its value as a feasibility study. Even with a relatively small sample size, an appropriate analysis technique was used to evaluate clinical changes in the participants. Although current study lacks a sham control to compare the effectiveness of DTx, our findings hold some tangible improvements in clinical measures, indicating that current study holds solid evidence over previous case control studies (35, 53). Second, a formal IQ evaluation was not performed during the preliminary evaluation. However, during the screening process, a child-adolescent psychiatrist evaluated intellectual disability. Third, it can be difficult to interpret the results independent of the medication effects. However, this study protocol specifically included only children who maintained the same dosage and were not receiving other medications except ADHD medications, such as antidepressants and anxiolytics. Fourthly, the current study did not gather any background information about the parents. Lastly, because a significant number of self-reported evaluations have been performed to analyze the effect, the placebo effect must be considered. Despite specific challenges, our study aimed to maintain a reasonable level of participant compliance despite the absence of financial incentives.

In conclusion, our findings suggest that there are tangible differences in DTx, such as NUROW, in children with ADHD, particularly in alleviating the clinical symptoms, including inattentiveness and hyperactivity reported by parents. As this study aimed to evaluate the potential efficacy and feasibility of NUROW, the findings provide preliminary evidence for the potential effects of DTx in improving attentional aspects and objective subclinical symptoms in children with ADHD. It is necessary to conduct well-designed research with a large sample size to analyze the effect of DTx.

## Data availability statement

The raw data supporting the conclusions of this article will be made available by the authors, without undue reservation.

## Ethics statement

The studies involving human participants were reviewed and approved by the Institutional Review Board of Chungnam National University Sejong Hospital (Approval Number: 2020-090038-007). Written informed consent to participate in this study was provided by the participants’ legal guardian/next of kin.

## Author contributions

TS, H-JK, and C-HC conceived and designed the study and performed statistical analyzes. TS, JY, H-JL, H-JK, and C-HC wrote the first draft of the manuscript. TS, K-YC, J-LK, H-JK, and C-HC participated in data collection. C-HC has full access to all the data in the study and is responsible for the integrity of the data and the accuracy of the data analysis. All authors edited all versions of the manuscript and were involved in interpreting the results and read, commented on, and approved the final version of the manuscript.

## Funding

This work was supported by National Research Foundation (NRF) of Korea grants funded by the Ministry of Science and Information and Communications Technology (MSIT), Government of Korea (NRF-2020R1C1C1007463 and NRF-2021R1A5A8032895), Information and Communications Technology (ICT) and Future Planning for Convergent Research in the Development Program for R&D Convergence over Science and Technology Liberal Arts (NRF-2022M3C1B6080866), and Institute of Information and communications Technology Planning and Evaluation (IITP) grant funded by the Korea government (MSIT) (No. RS-2023-00224823). This work was supported by a grant from Korea University (K2225691).

## Conflict of interest

The authors declare that the research was conducted in the absence of any commercial or financial relationships that could be construed as a potential conflict of interest.

## Publisher’s note

All claims expressed in this article are solely those of the authors and do not necessarily represent those of their affiliated organizations, or those of the publisher, the editors and the reviewers. Any product that may be evaluated in this article, or claim that may be made by its manufacturer, is not guaranteed or endorsed by the publisher.
