# Supplementary material for: Potential effectiveness of digital therapeutics specialized in executive functions as adjunctive treatment for clinical symptoms of attention-deficit/hyperactivity disorder: a feasibility study
Source: Front Psychiatry. 2023 Jul 20;14:1169030. doi: 10.3389/fpsyt.2023.1169030 (PMC10397734; doi:10.3389/fpsyt.2023.1169030)
Supplement: Supplementary file 1 [file Data_Sheet_1.PDF]

Supplementary Figures

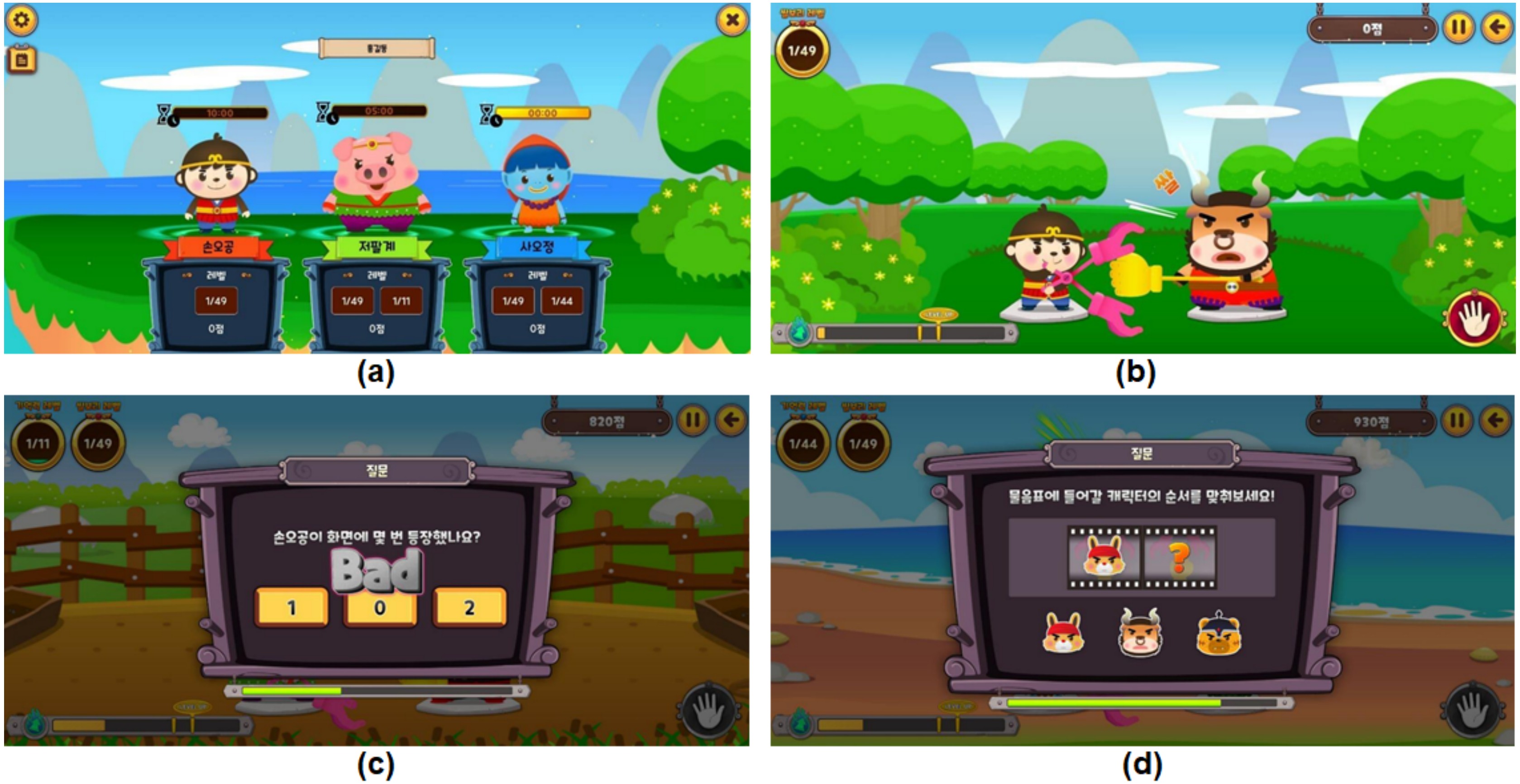

Supplementary Figure 1. Smartphone application images of digital therapeutic (NUROW) on Korean children with ADHD
(a) Screen image of training module selection. (b) *Sun Wukong* or *Monkey King* (Go/No-go task) training module is presented by advising users to tap on the screen (catching the yellow fist) when a certain signal is given. (c) *Zhu Bajie* or *Pigsy* (N-Back/Updating) training module is shown by asking users to answer how many times a certain character or picture (signal) appears on the screen while catching the fist. (d) *Sha Wujing* or *Sandy* (working memory) training module is presented by asking the users to memorize the sequence of the character images.
ADHD: Attention Deficit/Hyperactivity Disorder
